# Evaluating the impact and acceptability of a mental health literacy course for UK university students: a qualitative study

**DOI:** 10.1136/bmjopen-2026-123063

**Published:** 2026-09-22

**Authors:** Alexandra Newbold, Ellen Marshall, Frances Mathews, Edward Watkins, Anne C Duffy, Lucy Jayne Robinson, Katheryn F Morse

**Affiliations:** 1 Department of Health and Community Sciences, University of Exeter, Exeter, UK; 2 School of Psychology, Newcastle University, Newcastle upon Tyne, UK; 3 Department of Public Health and Sports Sciences, University of Exeter, Exeter, UK; 4 School of Psychology, University of Exeter, Exeter, UK; 5 Department of Psychiatry, Queen’s University, Kingston, Ontario, Canada; 6 University of Oxford Department of Psychiatry, Oxford, UK

**Keywords:** QUALITATIVE RESEARCH, Health Literacy, MENTAL HEALTH, EDUCATION & TRAINING (see Medical Education & Training)

## Abstract

**Abstract:**

**Objectives:**

To explore students’ experiences of engaging with an asynchronous, elective, digital mental health literacy (MHL) course adapted for delivery within UK higher education settings. Specifically, the study examined the perceived impact on MHL and acceptability. To identify recommendations for future course development.

**Design:**

Qualitative study using focus groups. Focus groups were audio-recorded, transcribed verbatim and analysed using Framework Analysis.

**Setting:**

Two universities (University of Exeter and Newcastle University).

**Participants:**

Nineteen undergraduate students predominantly studying psychology and social science subjects participated in one of five focus groups.

**Results:**

Four themes were identified from students’ experiences of the course, including a greater understanding of mental health, greater self-awareness and understanding of others experiencing mental health difficulties. Students perceived the course as relevant, useful and accessible, and provided recommendations for future course development. Students also highlighted a need for greater guidance on responding to mental health difficulties in others, including emotional boundaries, crisis response and appropriate signposting to professional support.

**Conclusion:**

Our findings suggest that MHL interventions may support greater understanding of well-being and mental ill-health, greater self-reflection and better understanding of the mental health of others within university settings. Students perceived the MHL course as relevant, acceptable and beneficial but identified uncertainty regarding how to safely respond to peers experiencing mental health difficulties. Future MHL interventions need to consider how to help students safely apply MH knowledge within their everyday interactions with peers and wider support networks.

**Strengths and limitations of this study**

- Multiple focus groups were held across two geographically contrasting universities increasing the diversity of student perspectives and experiences.
- Framework Analysis supported systematic comparison of perspectives across focus groups and enhanced transparency during data analysis.
- The qualitative design enabled detailed exploration of student experiences, acceptability and perceived engagement with the mental health literacy course.
- Inclusion of optional in-person and digital course components across two UK university sites enabled exploration of differing student preferences regarding course delivery and support.
- Participants were self-selecting and predominantly female psychology and social science students, which may limit transferability of findings to broader student populations.

## Introduction

 The proportion of university students who report a mental health concern is increasing, with 0.7% of students reporting a mental health condition in 2010/2011 rising to 4.5% in 2021–2022.^1^ University students may experience a range of pressures related to academic performance and social integration,^2^ as well as concerns about future career prospects and broader psychosocial stressors associated with the transition to higher education.^3^ Increasing demand for support means that university mental health and well-being services, alongside wider National Health Service (NHS) mental health provision, often struggle to meet student need, with many students experiencing long wait times for counselling and psychological support.^4^ These challenges highlight the need for proactive and preventative approaches to student mental health support, including the development, implementation and evaluation of early intervention initiatives.^5^

One promising mental well-being approach for students has been the promotion of mental health literacy (MHL) tailored to university student concerns. MHL has been conceptualised into four major dimensions: knowledge of how to obtain good mental health, understanding what constitutes poor mental health, reducing stigma and improving help-seeking.^6^ MHL courses can equip learners with knowledge and skills to support both their own well-being and that of others while reducing stigma associated with mental health difficulties.^7–9^ These interventions also enhance the ability of students to recognise mental health issues in themselves and others, facilitating earlier identification and response to emerging difficulties.^10 11^ However, increased awareness of mental health difficulties among peers may also create uncertainty around best practice responses, particularly in relation to emotional boundaries, crisis response and signposting to professional support.^12^ Mental health education may facilitate earlier recognition of difficulties and timely help-seeking,^13 14^ so students may benefit from improved understanding of mental health and well-being, including healthy coping strategies, symptom recognition and knowledge of how, when and where to seek support.^15^ Embedding MHL within university curricula may support resilience, emotional self-awareness and mental health understanding.^16^

MHL interventions delivered to university students vary considerably in their goals, content, duration and delivery format,^17 18^ and this can contribute to a lack of consensus on best practice. Common course components include psychoeducation on specific mental health disorders such as depression and anxiety, alongside strategies for stress management, resilience and help-seeking.^17^ Some courses incorporate testimonials or lived-experience narratives that may reduce stigma and/or improve empathy. Interactive elements, including quizzes and videos, have also been found to enhance student engagement and retention of knowledge.^19^ Recent recommendations emphasise flexible and accessible digital course design that accommodates diverse learner needs.^20^

The reported outcomes of MHL courses vary according to course structure, content and their target population.^17^ Evaluations of MHL have reported improvements in recognition of mental health difficulties, stigma reduction and confidence in responding to mental health challenges.^21 22^ Compared with a control group of non-course takers, course participation has been associated with improvements in broader well-being indicators, including sleep quality and substance use.^21^ The course evaluated in the present study was developed using principles derived from the Social Cognitive Theory (SCT),^23^ with content designed to increase mental health knowledge, self-awareness and confidence in recognising mental health difficulties. However, quantitative evaluations alone provide limited insight into how students experience MHL courses, which course components are most valued, and what factors influence engagement and acceptability.

### Research gap, aims and objectives

Despite growing research into MHL courses, relatively little is known about how best to tailor these courses to meet the specific needs of university students and ensure their acceptability and meaningful engagement.^17 24^ Qualitative methods, including focus groups, enable exploration of how students experience and interpret MHL content within real-world educational settings. Such approaches inform course design and implementation from the student perspective. The present study aimed to explore students’ experiences of engaging with a university MHL course adapted for delivery in UK higher education settings. Specifically, the study sought to:

1. Explore students’ perceptions of the course’s impact on MHL.
2. Examine factors influencing acceptability and engagement.
3. Identify barriers to engagement and recommendations for future course development

This research seeks to strengthen understanding of how students experience MHL interventions, and to inform their implementation, refinement and delivery within university settings.

## Method

This study is a qualitative evaluation of the MHL course piloted across two universities in England, the University of Exeter (UoE) and Newcastle University (NU). Pre- and post-course quantitative survey results of the MHL course are being reported elsewhere.^25^ To support the context of this evaluation, we provide a brief outline of the MHL course used.

### The U-flourish adapted UK MHL course

The MHL course evaluated was a contextually and culturally adapted version of the accredited digital Canadian MHL course ‘The Science of Wellbeing, Mental Health and Resiliency’ developed as part of the U-Flourish Student Mental Health Research programme.^21^ The original U-Flourish course was informed by SCT,^23^ which proposes that learning and behaviour are shaped through interactions between knowledge, beliefs, self-efficacy and social influences. Consistent with SCT, the course aimed to improve mental health knowledge, increase self-awareness, reduce stigma and enhance confidence in recognising and responding to mental health difficulties in oneself and others. It was developed using state-of-the-art online pedagogical and equity and inclusion principles and produced using reverse mentorship in partnership with undergraduate students. The course was designed as a relevant, evidence-informed and engaging educational course, rather than a mental health first aid course. Accordingly, this course focused on improving MHL, emotional self-awareness, stigma reduction and health-related behaviours. To support learner engagement, the course incorporated a range of learner activities, including embedded formative reflections, written narratives, brief audio and video presentations by experts, and fictional student character vignettes to support accessibility and engagement.

The U-Flourish MHL course was adapted for the UK context and piloted at Exeter and Newcastle Universities. The course consisted primarily of an asynchronous digital MHL course, supplemented by optional in-person support and reflection sessions at both universities. The course was designed to be completed across six modules comprising 120 hours of learning content consistent with full-term accreditation requirements. Although modules were released sequentially, students progressed at their own pace. Course implementation varied between sites (table 1). While the core online MHL content was consistent across universities, it was embedded differently within local curricula. Newcastle students completed the course as a credit-bearing component of their studies and participated in structured, tutor-supported reflection activities, whereas Exeter students undertook the course on a voluntary, non-credit-bearing basis and were offered optional support sessions.

**Table 1:** Cross site summary of the MHL course delivery variation

Site	Discipline	Degree stage	Year of participation	Assessment	Incentive for completion
NU	Combined honours students in the faculty of humanities.	Undergraduates,	2022–23	Reflective diary	10 credits
	Social science, medical science and psychology	First years only	2023–24	Personal well-being plan	
UoE	Psychology	Undergraduates	2022–23	Short online test at end of MHL course	Certificate of attendance

MHL, mental health literacy; NU, Newcastle University; UoE, University of Exeter.

### The focus groups

#### Participants

Eligible participants from NU and UoE were predominantly studying psychology and social science subjects and had chosen to enrol on the Nurture-U MHL course. They were invited via email and had 2 weeks to respond. Eligible participants were told that we were looking for feedback on the MHL course, including ways that it could be improved and that they did not need to have finished the course to attend.

NU: researcher EM ran four focus groups, two in December 2022 and two in December 2023 with a total of 14 female undergraduate students who were paid £10 cash for attending and received food while at the focus group.

UoE: researcher AN ran one focus group with five female undergraduate students in April 2023. Students were paid £20 in shopping vouchers for participating in the focus group.

### Data collection

A series of focus groups were run to collect information from students’ experiences and perceptions of engaging with the MHL course. The discussion guide was developed by members of the Newcastle research team (EM and LR) to obtain feedback across all aspects of the course, including practical features, participant experiences, perceived impacts on mental health and well-being, and opportunities for both positive and negative feedback, as well as suggestions for future improvement. The guide was used at the first focus group and based on participant feedback and the first stages of analysis, the research team determined the questions were sufficient and covered the topics required for evaluation. Therefore, the same interview guide was used for all subsequent focus groups. A copy of the discussion guide is provided in online supplemental appendix A. Semi-structured questions covered a range of topics, including participant reflections on being a participant in the course, the volume and type of content, positive and negative experiences of the course and the impact the course had. Students were provided with an information sheet describing the study and were required to complete and return a consent form prior to attending a focus group. Participants were remunerated for their time following completion of the focus group discussion.

Focus groups lasted approximately 1 hour. Interviewers were qualitative mental health researchers (AN and EM), experienced in running focus groups and were unknown to the students. Students were encouraged to give open and honest feedback. Researchers explained the purpose of the focus groups was to collect views from students who had taken the MHL course in order to inform, amend and improve future delivery. Only the two researchers and transcribers had access to the voice files.

### Analysis

We selected Framework Analysis for analysing data by organising transcribed qualitative data into charts or tables, which allowed its organisation into themes.^25^ Framework Analysis is frequently used in applied healthcare research, including studies exploring mental health experiences and service evaluations of young people.^26–28^ Researchers AN and EM followed the five steps of Framework Analysis^29^: (1) first they familiarised themselves with the transcript before developing an initial coding framework that combined a priori topics from the discussion guide with inductive concepts emerging from the data; (2) the framework was reviewed and refined collaboratively before being used to (3) index and chart data into themes and subthemes; (4) researchers then iteratively reviewed and recoded data extracts to ensure adequate representation across themes and (5) findings were subsequently summarised and interpreted collaboratively.

Both researchers had professional and research interests in student mental health, MHL and preventative approaches to well-being. They recognised that these backgrounds could have shaped interpretation of participant accounts, including assumptions that MHL is beneficial for undergraduate students, that increasing mental health knowledge and self-awareness can support well-being, and that universities have a role in providing preventative mental health education. Neither researcher was involved in participants’ teaching, was known to participants prior to the focus groups, or was involved in the development or delivery of the course. This provided the researchers with some analytical distance from the course. Reflexive discussions were undertaken throughout the analysis to identify and challenge these assumptions, consider alternative interpretations and ensure that findings remained grounded in participants’ accounts, including views that questioned the value, relevance or acceptability of the course.

### Patient and public involvement

This work is a part of the Nurture-U project (https://www.nurtureuniversity.co.uk/) which has a significant student involvement element. Student involvement included reviewing and providing feedback on the course content and wording, informing adaptation of the course for a UK university context and contributing feedback through the present evaluation.

### Standards for Reporting Qualitative Research

We used the Standards for Reporting Qualitative Research (SRQR) reporting guideline^30^ to draft this manuscript, and the SRQR reporting checklist^31^ when editing, included in online supplemental A. The study was conducted within an applied qualitative research framework informed by a pragmatic approach focused on understanding student experiences and informing course development. While pragmatism informed the methodological orientation of the study, the course itself was developed using principles derived from the SCT.^23^ The SCT informed the conceptual understanding of how participation in the course may influence students’ MHL, self-awareness and confidence in recognising and responding to mental health difficulties. The interpretation of findings relating to course acceptability was subsequently informed by the Theoretical Framework of Acceptability (TFA).^32^ The TFA provided a useful lens for understanding factors influencing students’ perceptions of relevance, usefulness, engagement and burden associated with the course.

## Results

Four themes and eleven subthemes were identified describing students’ perceptions of the impact, acceptability and future development of the MHL course. The themes and sub-themes are listed below in table 2.

**Table 2:** Table of themes and sub-themes

Themes	Sub-themes
1. Perceived impact of the course	1.1 Understanding mental health 1.2 Understanding yourself and others
2. Acceptability and engagement	2.1 Relevance and usefulness of the course 2.2 Valuable content and learning approaches 2.3 Flexibility of online delivery 2.4 Discussion and reflection opportunities
3. Barriers to engagement and acceptability	3.1 Volume and complexity of content 3.2 Tailoring content to differing levels of prior knowledge 3.3 Navigation, usability and technical challenges
4. Recommendations for future course development	4.1 Missing content areas 4.2 How to safely help others

### Theme 1: perceived impact of the course

#### Understanding mental health

Students described how participation in the MHL course increased their awareness and understanding of mental health and well-being, including factors influencing mental health difficulties in themselves and others.

It’s made me definitely more just aware in general. (FG1, P1)

Students particularly valued learning about the science underpinning mental health. Understanding biological and genetic influences was described as validating.

It felt validating, of like ‘okay so it isn’t just I’m strange’… I liked the science… that you’re not the problem. (FG1, P5)

#### Understanding yourself and others

Students described how the course encouraged greater reflection, self-awareness and self-compassion.

It made it easier to realise, if you’re having a bad day, you’re not on your best form, and that’s also okay. (FG1, P1)

Students described how participation in the course improved their understanding of others experiencing mental health difficulties.

It made me, like click a bit better….they've gone into their room because they're like, having a bad day, like it’s not anything on you. Or you can like check in on them, like it just made me more understanding. (FG1, P2)

Students reflected on how learning about mental health reduced the tendency to misinterpret the actions of others and encouraged greater empathy.

### Theme 2: acceptability and engagement

Students viewed the MHL course positively and described it as relevant, useful and accessible.

#### Relevance and usefulness of the course

Students generally felt that the content was relevant to their experiences as university students. Students valued that the course focused on issues commonly encountered during university life.

It’s something that is relevant right now, every topic is really focused on us and what can affect us. (FG2, P3)

Students felt the course addressed aspects of mental health not routinely discussed within their academic programmes.

#### Valuable content and learning approaches

Topics relating to substance use, addiction and sleep were viewed as particularly relevant to student life.

They aren’t aware of the short-term or long-term effects it can have on them. I think to be informed on that is necessary. (FG2, P4)

Students also valued content explaining the science underpinning behaviour and well-being.

Just saying it’s bad for you doesn’t really resonate with anyone. (FG1, P1)

Case studies were also highlighted as effective learning tools that helped contextualise information.

I did really enjoy the case studies… I resonated with some of the things that they were talking about. (FG5, P2)

#### Flexibility of online delivery

Students valued the flexibility offered by the asynchronous online format. Being able to engage independently and at their own pace enabled participation around existing commitments.

It was nice to kind of just sit there and do it, by yourself and kind of think about how it related to yourself. (FG1, P4)

The online format also provided opportunities for reflection on sensitive topics.

In person I feel like you might withdraw a little. (FG1, P3)

#### Discussion and reflection opportunities

Students expressed differing preferences regarding discussion and reflection. Some valued the optional face-to-face sessions and described them as supportive environments for reflection.

I really liked the informality. (FG2, P5)

Others preferred to engage with the material independently and expressed reluctance to discuss mental health-related topics within a group setting. Concerns about privacy and personal disclosure influenced willingness to participate in discussion-based activities.

I don’t know if I want people to know everything about me. (FG5, P2)

Students suggested that opportunities for reflection should remain optional.

### Theme 3: barriers to engagement and accessibility

#### Volume and complexity of content

Students frequently described the course content as extensive and, at times, overwhelming. While they valued the breadth of information available, some felt that the volume of content reduced engagement and obscured key messages.

I do think that it was very wordy… at times I felt like I was kind of getting lost in a sea of words. (FG5, P3)

While some appreciated the depth of information provided, others felt that certain sections contained more detail than was necessary to understand key concepts.

It was a lot probably like of science that you didn’t really need to know to understand the concept. (FG1, P1)

#### Tailoring content to differing levels of prior knowledge

While many welcomed the introductory nature of the content, some psychology students would have valued opportunities to engage with more advanced content.

You already knew the things. (FG1, P3)

#### Navigation, usability and technical challenges

Students described difficulties navigating some aspects of the platform and suggested that features such as progress tracking, clearer course structure and improved navigation tools would enhance user experience.

Have a line at the bottom with all the slides… and see an overview of what each slide is. (FG5, P2)

Some students experienced technical difficulties, including problems saving progress and revisiting completed activities.

After I lost all my progress and stuff, I just kind of accepted that I wouldn’t get it. (FG5, P3)

### Theme 4: recommendations for future course development

Students identified several opportunities to strengthen future versions of the MHL course.

#### Missing content areas

Students identified several areas that they believed required greater attention. Eating disorders were highlighted as a notable omission. Students felt that increasing awareness of eating disorders would improve their ability to recognise potential signs and symptoms and when support may be needed.

Being able to recognise that is really important because a lot of signs are obvious when you know them, but very invisible when you don’t. (FG5, P1)

Students also suggested including additional information about support options and recovery experiences to improve the practical relevance of the course.

These are the people you can talk to, and these are the resources you can seek, or even just like, these are the experiences people have had who have come through it and come out the other side. (FG1, P3)

#### How to safely help others

Students identified a need for greater guidance on responding to peers experiencing mental health difficulties, and described uncertainty regarding how to translate this knowledge into appropriate action.

It’s good to be knowledgeable, but… it’s not your responsibility to fix someone. (FG5, P3)

Students wanted practical guidance for responding to significant distress, self-harm, substance use or mental health crises. They described uncertainty regarding where responsibility for supporting peers should begin and end, particularly in situations involving risk or worsening mental health.

I’ve seen people that have taken bad drugs and were, couldn’t communicate, I don’t know how to take care of them… and at what point do we need someone. (FG5, P1)

Students suggested that future MHL courses should provide clearer guidance regarding emotional boundaries, crisis response and signposting to professional support services.

## Discussion

Students perceived the MHL course as a relevant and acceptable approach to improving the understanding of mental health within a university setting. Consistent with previous evaluations of MHL courses, they described increased understanding of mental health, themselves and others following participation.^17–19^ Students’ accounts support conceptualisations of MHL as extending beyond knowledge acquisition to encompass attitudes, beliefs and competencies relating to mental health.^10 11^ Students described greater self-reflection, self-compassion and understanding of others, suggesting that engagement with MHL content influenced how they interpreted and responded to mental health experiences within their own lives and social relationships. This supports previous research highlighting the potential for MHL courses to contribute to broader psychosocial outcomes and preventative approaches within university settings.^13 16 17^

These findings are broadly consistent with the SCT,^23^ which informed the development of the course. These students described not only acquiring knowledge about mental health but also applying it to themselves and others. The progression evident across the findings—from understanding mental health to understanding oneself and others—reflects the educational aims of the course and broader conceptualisations of MHL as encompassing knowledge, attitudes and competencies relating to mental health.^10 11^

A particularly important finding was the desire among students for greater guidance regarding how to support peers experiencing mental health difficulties. While they reported increased understanding of others and greater willingness to offer support, they also described uncertainty regarding emotional boundaries, responsibility, safeguarding and signposting decisions. These findings align with previous research, indicating that university students often report uncertainty regarding recognising and responding appropriately to mental health difficulties in peers, including eating disorders.^12^ The findings suggest that recognising mental health difficulties and knowing how to respond to them may represent distinct educational needs and that future MHL courses may benefit from greater emphasis on crisis response, signposting guidance and peer-support boundaries.

Findings relating to acceptability can be understood through the TFA.^32^ Students generally perceived the course as relevant, useful and appropriate for university students and valued the flexibility offered by the online format. However, perceived burdens such as excessive content, navigation difficulties and differing levels of prior knowledge influenced engagement for some. These findings are consistent with wider research highlighting the importance of usability, accessibility and engagement in digital mental health courses.^18 20 24^ Feedback from students also suggests that implementation factors such as usability, navigation, pacing and progress tracking may substantially influence engagement with digital MHL courses.

### Strengths and limitations

A key strength of this study is its reliance on rich qualitative feedback collected across two UK universities, providing insight into students’ experience of an MHL course, including its impact and acceptability. The use of focus groups enabled exploration of both shared and contrasting experiences of course engagement and participation. However, several limitations should be considered. The sample was self-selecting and may therefore have been biased towards students with a greater interest in mental health or more positive views of the course. These students were exclusively female, with the majority studying psychology or social science subjects, which may limit the transferability of findings to broader student populations. In addition, implementation differed across the two study sites. Newcastle students completed the course as a credit-bearing component of their programme and participated in structured tutor-supported reflection activities, whereas Exeter students engaged with the course on a voluntary non-credit-bearing basis. These differences may have influenced motivation, engagement and perceptions of the course. Finally, while recruitment did not aim to achieve data saturation in a positivist sense, consistent patterns across focus groups and the absence of substantially new concepts in later discussions suggested sufficient thematic depth to address the study aims.

### Implications for practice and future research

The findings suggest that university MHL courses may represent a valuable component of broader preventative approaches for student mental ill-health. Given increasing pressure on university counselling services and wider NHS mental health provision, scalable psychoeducational courses may support students’ MHL and awareness and understanding of well-being. Future MHL courses need to consider implementation and user experience alongside educational content. The findings from the present study provide evidence that students would value greater guidance on recognising when professional support is required, understanding signposting options, maintaining emotional boundaries and recognising when support may be needed for self or others. Further research, including longitudinal studies and randomised controlled trials, is needed to evaluate implementation, acceptability and effectiveness of MHL courses across diverse university populations.

## Conclusion

Students perceived the MHL course evaluated in this study as a relevant and acceptable approach within a university setting. Findings suggest that MHL courses may support important educational and preventative objectives within higher education. The designers of future MHL courses should consider not only what students need to know about mental health but how they can safely apply that knowledge within their everyday interactions and support networks.

## Supplementary material

10.1136/bmjopen-2026-123063 online supplemental file 1

10.1136/bmjopen-2026-123063 online supplemental file 2

## Data Availability

Data are available upon reasonable request.
