# Supplementary material for: Evaluating the impact and acceptability of a mental health literacy course for UK university students: a qualitative study
Source: BMJ Open. 2026 Sep 22;16(9):e123063. doi: 10.1136/bmjopen-2026-123063 (PMC13599973; doi:10.1136/bmjopen-2026-123063)
Supplement: Supplementary file 1 [file bmjopen-2026-123063.reviewer_comments.pdf]

PEER REVIEW HISTORY

BMJ Open publishes all reviews undertaken for accepted manuscripts. Reviewers are asked to complete a checklist review form and are provided with free text boxes to elaborate on their assessment. These free text comments are reproduced below.

ARTICLE DETAILS

Title (Provisional)

Evaluating the Impact and Acceptability of a Mental Health Literacy Course for UK University Students: A Qualitative Study

Authors

Newbold, Alexandra; Marshall, Ellen; Mathews, Frances; Watkins, Edward; Duffy, Anne C; Robinson, Lucy Jayne; Morse, Katheryn F

VERSION 1 - REVIEW

**Reviewer**	**1**
**Name**	**Rees, Susan**
**Affiliation**	**University of New South Wales Medicine & Health, Psychiatry and Mental Health**
**Date**	**10-Jun-2026**
**COI**	**None**

Thank you for the opportunity to read and review this paper, I think it makes a valuable contribution to the field.

Overall comment.

It is a well-conducted applied qualitative evaluation addressing an important issue in student mental health. The manuscript is clearly written, the rationale is sound, and the findings provide practical recommendations for improving mental health literacy (MHL) interventions in higher education. The use of Framework Analysis is appropriate for the evaluation aims. However, several methodological and conceptual issues should be addressed before publication.

Major comment

The study lacks a clearly articulated informing theory. The authors state that the study was conducted within an "applied qualitative research framework informed by a pragmatic approach." While pragmatism provides a methodological orientation, it is not a substantive theory explaining how students engage with, interpret, and apply MHL content.

The absence of an explicit theoretical framework weakens interpretation of the findings and limits conceptual contribution beyond description.

A suggested theory could be Acceptability of Healthcare Interventions Framework (Sekhon et al.) or also Normalisation Process Theory could help explain how students incorporate MHL knowledge into everyday behaviours and peer-support practices. Another thought, from an educational perspective is Social Cognitive Theory which could explain reported changes in self-awareness, self-compassion, help-seeking confidence, and peer-support behaviours.

The assessment of student acceptability of the MHL course outcome has been achieved, as has the exploration of students' experiences of engaging with the course and its perceived influence on MHL and help-seeking. The paper also succeeds in identify challenges and suggestions for improving the course.

Some points: The experiences of engagement are well covered (participant experiences, self-compassion, understanding others, peer support). However, the objective also refers to help-seeking. Evidence regarding actual or intended help-seeking is relatively limited. The findings focus more on awareness and attitudes than on help-seeking processes or behavioural intentions. Greater analytic attention to help-seeking would strengthen alignment with the stated objective.

The themes "General Suggestions for Improving Content," "Missing Content," "Course Formatting and Navigation," and "Course Delivery and Support" directly address this objective and provide actionable recommendations.

Methodological Issues

The reflexivity section is brief and more explicit reflexive positioning would strengthen methodological transparency.

The authors note their professional interests in student mental health but provide little detail regarding their own positioning, for example what are their backgrounds, their assumptions about MHL, their relationship to the intervention or how their positioning may influence the interpretation of results (this is important for qualitative research).

The authors refer to "sufficient thematic depth" rather than saturation. This is acceptable, but a more detailed explanation of how thematic sufficiency was judged would improve rigour.

Reviewer 2

Name Philippot, Arnaud

Affiliation KU Leuven, Research Group for Adapted Physical Activity and Psychomotor Rehabilitation

Date 16-Jun-2026

COI **None**

Thank you for the opportunity for reviewing your manuscript entitled "Evaluating the Impact and Acceptability of a Mental Health Literacy Course for UK University Students: A Qualitative Study" (bmjopen-2026-123063) to BMJ Open.

After consideration, I regret to inform you that I suggest to reject this manuscript for publication in its current form. Several significant concerns that, taken together, prevent from moving forward at this stage such as the need for more concise introduction, results and discussion, no-use of framework for evaluating the training, a required more structured results part revealing the most relevant insights.

I would like to close by acknowledging the importance of the work you have done. Research on mental health literacy in university students addresses a genuinely pressing public health issue, and the effort involved in designing, piloting and evaluating such a course across two university sites is commendable. We hope the feedback provided will help strengthen this work, and we look forward to seeing it published in due course.

Here some additional comments:

ABSTRACT

- The type of students targeted by the course should be specified more precisely (e.g., discipline, age).

- The first lines of the conclusion ("Qualitative analysis of student feedback identified several factors influencing the acceptability and perceived usefulness of the MHL course...") read as results rather than conclusions and should be revised accordingly.

GENERAL

- The manuscript is substantially too long in its current form. The introduction could be shortened considerably. Qualitative data should be better synthesised, and we strongly suggest presenting the main themes alongside illustrative verbatims in a summary table to alleviate the volume of text.

TABLE 1

Furthermore, the introduction states that the course is non-credit bearing, yet Table 1 indicates that credit is awarded at one site, this inconsistency must be resolved. A legend defining NU and UoE should also be added and Undergraduate students are not consistently written. Age could be added.

- The paragraph describing the in-person support sessions at Newcastle and Exeter is confusing, as it is initially unclear whether these sessions refer to mental health support or course-related academic support. As it does not add substantial information, we recommend deleting it.

- The manuscript currently lacks a structured theoretical framework to organise and deepen the analysis with a mixed approach. Existing frameworks such as the Kirkpatrick Model (https://www.kirkpatrickpartners.com/the-kirkpatrick-model/) would provide a more rigorous and structured basis for evaluating the course's impact and would substantially strengthen the contribution of the paper.

RESULTS

- The results section, like much of the manuscript, lacks conciseness. Sub-themes 1.1, 1.2 and 1.3 should be merged into a single section, as 1.1 does not provide sufficiently distinct or relevant insights to stand alone.

VERSION 1 - AUTHOR RESPONSE

BMJ Open Revision Cross-Reference Table

**Reviewer/Editor Comment**	**Action Taken**
The type of students targeted by the course should be specified more precisely (e.g., discipline, age).	Participant characteristics were clarified in the Abstract and Methods. The participant sample is now described as 'female undergraduate students predominantly studying psychology and social science subjects. Age data were reviewed but were not available for all participants and therefore weren't reported. Location: Abstract (Revision 1, from line 17); Methods – Participants, (from line 233).
The first lines of the conclusion read as results rather than conclusions and should be revised accordingly.	The Abstract conclusion was revised to focus on interpretation, implications and future course development rather than repeating findings. Location: Abstract (Revision 1, from line 35).
The manuscript is substantially too long in its current form. The introduction could be shortened considerably.	The manuscript has been shortened from 4930 to 3909 words to improve conciseness, remove repetition and strengthen focus on the rationale for the study. Location: Introduction (changes from line 79).
The assessment of student acceptability of the MHL course outcome has been achieved, as has the exploration of students' experiences of engaging with the course and its perceived influence on MHL and help-seeking. However, evidence regarding actual or intended help-seeking is relatively limited. Greater analytic attention to help-seeking would strengthen alignment with the stated objective.	The study aims were revisited and refined. One objective was revised to focus on mental health literacy and acceptability, rather than help-seeking, improving congruence between the intervention, qualitative data and findings. The importance of help-seeking was retained within the Discussion and expanded within the Implications for Practice and Future Research section, including recommendations relating to recognising when professional support is needed, referral pathways and future intervention development. Location: Research Gap and Aims (Revision 2, from line 156); Implications for Practice and Future Research, (Revision 9, from line 780).

Please provide more detail about the interview/discussion guide. How was it created? Who was involved? Was it pilot tested? Why were these questions selected?	Information was added describing who developed the discussion guide, why questions were selected, how the guide was reviewed following the first focus group, and why no substantive revisions were required before use within subsequent focus groups. Location: Methods – Data Collection (Revision 5, line 246).
The absence of an explicit theoretical framework weakens interpretation of the findings and limits conceptual contribution beyond description.<br><br>The study lacks a clearly articulated informing theory. While pragmatism provides a methodological orientation, it is not a substantive theory explaining how students engage with, interpret and apply MHL content.	Social Cognitive Theory (SCT) informed the development of the intervention and is now used in the interpretation of findings. This provided a framework for understanding how students described engaging with, interpreting and applying MHL content within their own lives and relationships (Revision 3 from line 188)<br>The Discussion was substantially revised and shortened. Findings are now interpreted through SCT and TFA and situated within the wider MHL literature. Location: Discussion (Revision 9 from line 699).<br>The distinction between pragmatism as a methodological orientation and SCT as the intervention's informing theory has been clarified. Location: Introduction; Methods – U-Flourish Course Description (Revision 3, from line 188); SRQR (from line 329); Discussion (Revision 9, from line 699).
R2. The manuscript currently lacks a structured theoretical framework to organise and deepen the analysis. Existing frameworks such as the Kirkpatrick Model would provide a more rigorous and structured basis for evaluating the course's impact.<br><br>R1. A suggested theory could be the Acceptability of Healthcare Interventions Framework (Sekhon et al.), Normalisation Process Theory or Social Cognitive Theory.	The suggested frameworks were reviewed. SCT and TFA were selected because they provided the strongest alignment with the intervention, study aims and qualitative findings. Location: Methods – U-Flourish Course Description (Revision 3, from line 188); SRQR (Revision 7, from line 329); Discussion (Revision 9, from line 699)
The introduction states that the course is non-credit bearing, yet Table 1 indicates that credit is awarded at one site. This inconsistency must be resolved.<br>The paragraph describing the in-person support sessions at Newcastle and Exeter is confusing, as it is initially unclear whether these sessions refer to mental health support or course-related academic support. As it does not add substantial information, we recommend deleting it.	Course delivery was clarified to distinguish between Newcastle (credit-bearing) and Exeter (non-credit-bearing) implementation. The section was substantially shortened. Location: Methods – U-Flourish Course Description; Table 1 (from line 221); Course-related reflection opportunities (Revision 4, from line 212).
A legend defining NU and UoE should be added.	Definitions of NU and UoE were added beneath Table 1. Location: line 222
Undergraduate students are not consistently written.	Participant terminology was reviewed and standardised where appropriate. The term ‘students’

	is used throughout most of the manuscript, whereas undergraduate students is retained where participant characteristics are being described (e.g. Abstract (line 17), Methods, Table 1 (line 221).
The reflexivity section is brief and more explicit reflexive positioning would strengthen methodological transparency.<br>The authors note their professional interests in student mental health but provide little detail regarding their own positioning, assumptions about MHL, relationship to the intervention or how this may influence interpretation of results.	The reflexivity section has been expanded to include researcher backgrounds, professional interests, assumptions about MHL, analytical distance from the intervention to better describe the reflexive discussions undertaken throughout the analysis process. Location: Methods – Analysis (Revision 6, from line 303).
The results section, like much of the manuscript, lacks conciseness. Sub-themes 1.1, 1.2 and 1.3 should be merged into a single section.<br><br>Qualitative data should be better synthesised.<br><br>Qualitative data should be better synthesised, and we strongly suggest presenting the main themes alongside illustrative verbatims in a summary table to alleviate the volume of text.	The findings were re-organised from six into four overarching themes. The original themes 'Understanding yourself' and 'understanding others', were merged into a single subtheme, within the broader theme Perceived Impact of the Course to reduce fragmentation and improve synthesis.<br>Table 2 was revised to provide a clearer overview of the thematic structure. Quotations were retained within the narrative Results section to preserve context and support interpretation.<br>Repetitive interpretive statements were removed, related findings were consolidated into broader thematic groupings, and the Results section was substantially shortened to improve synthesis, whilst retaining illustrative participant quotes.<br>Location: Results (Revision 8, from line 340)
The themes General Suggestions for Improving Content, Missing Content, Course Formatting and Navigation, and Course Delivery and Support directly address this objective and provide actionable recommendations.	Related findings were re-organised into broader thematic groupings within the revised Results structure to improve synthesis whilst retaining participant recommendations and implementation insights. Location: Results (Revision 8 from line 340).
The authors refer to sufficient thematic depth rather than saturation. This is acceptable, but a more detailed explanation of how thematic sufficiency was judged would improve rigour.	Additional explanation was added describing thematic depth, consistency across focus groups, absence of substantially new concepts in later discussions and rationale for determining the data were sufficient to address the study aims. Location: Strengths and Limitations, (from line 756).
