# Supplementary material for: Evaluating the impact and acceptability of a mental health literacy course for UK university students: a qualitative study
Source: BMJ Open. 2026 Sep 22;16(9):e123063. doi: 10.1136/bmjopen-2026-123063 (PMC13599973; doi:10.1136/bmjopen-2026-123063)
Supplement: Supplementary file 2 [file bmjopen-16-9-s001.docx]

**Appendix A The Focus group questions**

The questions asked can be found in Table 3 below:

**Table 3: The questions asked in the focus groups**

| **Topic** | **Example questions** |
| --- | --- |
| Overall experience | - How did you find the course? - What factors did you consider when / what were your reasons for deciding to take this course? - What have you learnt about yourself as a person during this course? - What, if any, impact has this course had your day-to-day life? - Speaking to a friend, how would you describe the course to them? |
| Content | - Tell me about your opinions on the course content? - What content would you add/take out of the course? - What was your favourite topic included in the course? - Are there any topics you would like to add to the course? - Are there any topics you would like to remove? - Were there any elements that were particularly interesting /helpful? - Were there any elements that were redundant or not very helpful -e.g., covering things you already knew? - What did you think of the student vignettes? How did they add / effect your learning? |
| Format | - How did you find the online course format? - What your thoughts and experiences on the 3 drop-in sessions? - How did you fit / balance the course with other modules and commitments? - How would you feel about taking this course as part of your 120 credits? |
| Positives | - What part(s) of the course did you find useful? - What was the best thing about the course? - If you were going to, what aspects of the course would you talk about when recommending this to other students? |
| Challenges / difficulties | - If you could change one thing about the course, what would you change? And why? - What parts of the course did you not find as useful? And why? - What aspects would prevent you from recommending the course to other students? - What’s one thing you wish the course consisted of that it didn’t already? |
| Closing questions | - Are there any other points anyone would like to make about the course? - Is there anything else that wasn’t touched on that you think is relevant? |
